# Psoriasis flare following tirzepatide initiation in a patient with plaque psoriasis in remission: A case report

**DOI:** 10.1016/j.jdcr.2026.02.006

**Published:** 2026-02-11

**Authors:** Joan M. Neptune Rosa, Bianca Rivera Oquendo, Eneida De La Torre Lugo

**Affiliations:** a Department of Medicine, San Juan VA Caribbean Healthcare System, San Juan, Puerto Rico; b Department of Medicine, Ponce Health Sciences University, Ponce, Puerto Rico; c Department of Dermatology, University of Puerto Rico Medical Sciences Campus, San Juan, Puerto Rico

**Keywords:** adalimumab, Adverse drug reaction, Biologic therapy, GLP-1 receptor agonist, Immunometabolic therapy, Ixekizumab, Plaque psoriasis, Psoriasiform eruption, Psoriatic flare, Tirzepatide

## Introduction

Psoriasis is a chronic, immune-mediated inflammatory dermatosis affecting approximately 2% to 3% of the global population, characterized by erythematous, scaly plaques and a relapsing-remitting course. While disease flares are commonly associated with physical, environmental, and psychological stressors, infections, and certain medications, emerging evidence suggests that metabolic pathways and immunomodulatory therapies may also influence disease activity.^1^ Tirzepatide, a dual glucose-dependent insulinotropic polypeptide (GIP) and glucagon-like peptide-1 receptor agonist (GLP-1 RA), has rapidly gained widespread use for type 2 diabetes mellitus and weight management due to its potent metabolic benefits.^2^ While GLP-1-based therapies are generally well tolerated, they have occasionally been linked to diverse cutaneous reactions and immunologic effects.^3^ However, available data regarding their relationship with psoriatic disease remains limited and heterogeneous, with reports describing both clinical improvement and psoriasiform exacerbation.

Here, we describe the case of a patient with previously well-controlled plaque psoriasis who experienced a psoriatic flare after initiating tirzepatide.

## Case report

A 37-year-old female patient with a history of stable plaque psoriasis presented to the dermatology clinic with a 3-month history of a new, progressively pruritic generalized eruption. Her baseline plaque psoriasis had been in sustained clinical remission for more than 6 months. At the time of eruption onset, her medications included lisinopril/hydrochlorothiazide 10 mg/25 mg daily for hypertension, and topical metronidazole 0.75% cream twice daily for rosacea. Topical halobetasol 0.05% ointment had previously been prescribed for psoriasis on an as-needed basis but was not in active use.

**Fig 1:**
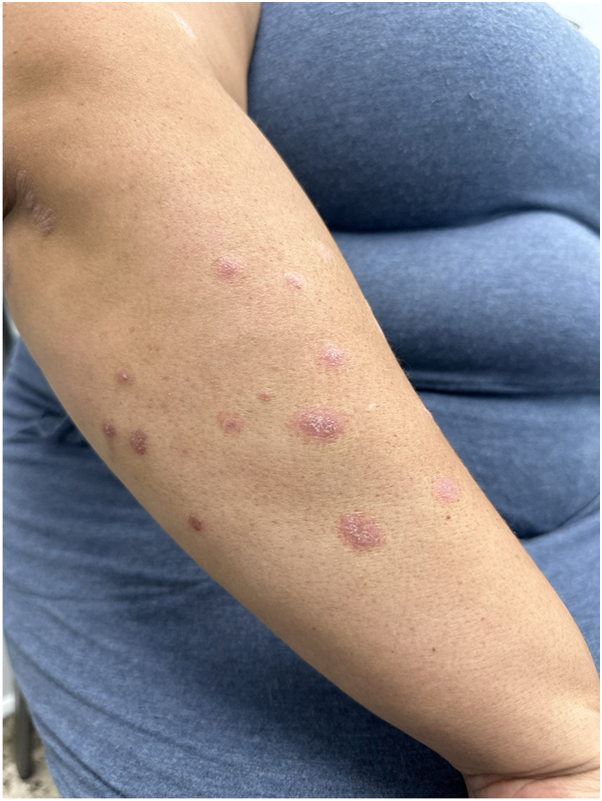
Psoriatic flare. Erythematous papules and small plaques with fine scale on the right dorsal arm at initial presentation.

**Fig 2:**
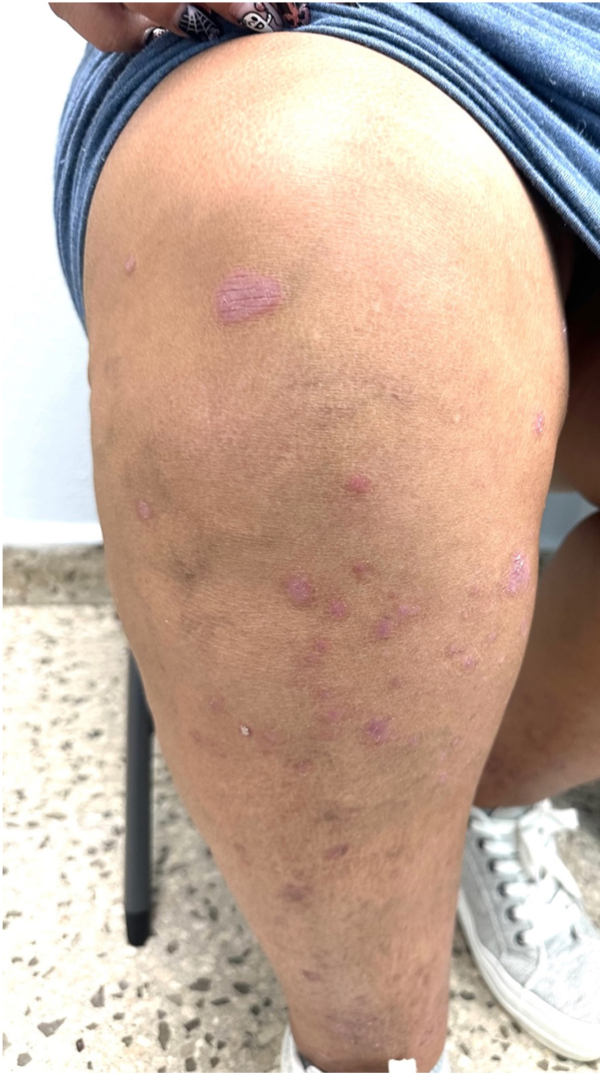
Psoriatic flare. Erythematous papules and small plaques with fine scale on the anterior right lower leg at initial presentation.

Physical examination revealed numerous well-demarcated erythematous papules and plaques measuring 3 mm to 1.5 cm, with overlying fine scale, scattered across the extensor surfaces of the forearms, anterior lower legs (pretibial/shin surfaces), and the trunk, consistent with an acute psoriasis flare (Figs 1 and 2). No pustules, vesicles, ulceration, or crusting were observed. A detailed history indicated that the eruption began approximately 4 weeks after the initiation of tirzepatide for weight loss, without preceding upper respiratory infection, pharyngitis, medication changes, or intercurrent illnesses.

Initial laboratory workup, including a complete blood count, comprehensive metabolic panel, C-reactive protein, and infectious screening tests such as a tuberculin skin test and hepatitis panel, was unremarkable. Formal streptococcal testing was not performed at the time of presentation. Skin biopsy was not obtained, as the diagnosis of psoriasis was made clinically based on the characteristic morphology and distribution of lesions in the context of a known history of psoriasis.

Oral methotrexate and topical halobetasol ointment 0.05% were started as first-line therapy. Given the strong temporal association, the eruption was attributed to the recent initiation of tirzepatide, prompting its discontinuation. However, at follow-up, lesions demonstrated a mixed clinical course with partial resolution of existing lesions alongside the emergence of new psoriatic papules and small plaques on the dorsal arms, knees, and shins over the subsequent 7 weeks.

**Fig 3:**
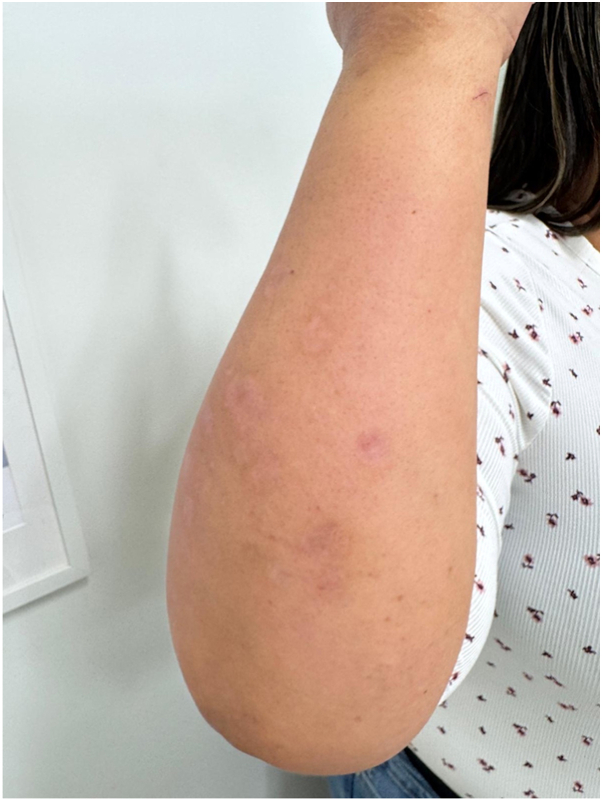
Psoriatic flare, resolving. Residual post-inflammatory pigmentary alteration on the dorsal forearm at follow-up, with resolution of previously noted erythematous papules and plaques following ixekizumab therapy.

Given persistent disease activity despite methotrexate and topical corticosteroid therapy, treatment was escalated to a biologic agent, adalimumab, leading to marked improvement and clearance over the next 3 months. Following the insurance-mandated transition to a biosimilar adalimumab formulation, disease activity recurred, prompting the initiation of ixekizumab, which resulted in notable improvement and early resolution of lesions over the following 3 months (Fig 3).

## Discussion

Preclinical and clinical evidence suggest that GLP-1 RAs may improve psoriatic disease through immunometabolic mechanisms, including reduced dermal inflammatory infiltration and modulation of cytokines central to psoriasis pathogenesis, such as tumor necrosis factor-α (TNF-α) and interleukins 6, 17, and 23.^4^^,^^5^ Tirzepatide, with its dual agonism, may further potentiate these pathways through its superior effects on weight reduction and glycemic control. Despite modest improvements in psoriasis severity index scores and symptoms such as pruritus,^5^ psoriasiform flares have been reported despite their anti-inflammatory profile.

In parallel, post-marketing surveillance data have identified a growing number of dermatologic adverse events associated with GLP-1 RAs. In a recent analysis, Ituarte et al reported that liraglutide was the most frequently implicated agent, followed by semaglutide and tirzepatide.^6^ Reactions included rash, pruritus, alopecia, and bullous pemphigoid, with increasing reports following wider clinical use, highlighting the need for continued pharmacovigilance.^6^

Individual case reports further illustrate the spectrum of cutaneous reactions associated with GLP-1 RA therapy. Nowowiejska et al described a 34-year-old woman with mild psoriasis who developed an exacerbation involving the face, scalp, and intertriginous areas following liraglutide initiation.^7^ Similarly, Bostan et al reported a 56-year-old woman with type 2 diabetes who developed widespread scaly plaques shortly after starting exenatide, with biopsy findings consistent with drug-induced psoriasiform dermatitis and subsequent improvement after drug discontinuation and targeted therapy.^8^ More recently, Cook and Selby described a tirzepatide-associated guttate psoriasiform eruption, which resolved following treatment with risankizumab-rzaa.^9^

When considered alongside these published observations, this case supports that GLP-1 RA initiation may be temporally associated with flares of preexisting psoriasis. In our patient, consistent clinical improvement with adalimumab followed by ixekizumab supports the diagnosis of psoriasis and underscores the central roles of tumor necrosis factor and interleukin-17A in the psoriatic inflammatory cascade.^10^

Limitations of this report include the absence of histopathologic confirmation, uncertainty regarding potential streptococcal involvement due to lack of formal testing, and the inability to establish causality beyond a temporal association between tirzepatide initiation and the observed psoriasis flare. Additional evidence from larger clinical studies will be needed to better define this relationship over time.

This case contributes to the evolving understanding of GLP-1 RAs-associated psoriasiform eruptions and highlights the importance of maintaining clinical vigilance when new or worsening skin disease develops in patients using these agents. Prompt recognition, ongoing assessment, and initiation of targeted immunomodulatory therapy remain essential for optimizing outcomes in this emerging clinical scenario.

## Conflicts of interest

Dr Eneida De La Torre reports serving as a speaker for Eli Lilly and Bristol Myers Squibb. No other relevant conflicts of interest are disclosed.
